# Coping with change in predation risk across space and time through complementary behavioral responses

**DOI:** 10.1186/s12898-018-0215-7

**Published:** 2018-12-20

**Authors:** Pierrick Blanchard, Christine Lauzeral, Simon Chamaillé-Jammes, Clément Brunet, Arnaud Lec’hvien, Guillaume Péron, Dominique Pontier

**Affiliations:** 1 Laboratoire Evolution et Diversité Biologique, CNRS, UMR 5174, Université Toulouse III Paul Sabatier, Toulouse, France; 2 Centre d’Ecologie Fonctionnelle et Evolutive, UMR 5175, Centre National de la Recherche Scientifique (CNRS), Université de Montpellier, Université Paul Valéry Montpellier, Ecole Pratiques des Hautes Etudes (EPHE), 1919 Route de Mende, 34293 Montpellier Cedex 5, France; 3 Laboratoire Biométrie et Biologie Evolutive, CNRS, UMR 5558, Université de Lyon, Université Lyon I Claude Bernard, Villeurbanne, France

**Keywords:** Vigilance, Habitat use, Prey, Predation risk, Predator, Behavior

## Abstract

**Background:**

Our picture of behavioral management of risk by prey remains fragmentary. This partly stems from a lack of studies jointly analyzing different behavioral responses developed by prey, such as habitat use and fine-scale behavior, although they are expected to complement each other. We took advantage of a simple system on the Kerguelen archipelago, made of a prey species, European rabbit *Oryctolagus cuniculus*, a predator, feral cat *Felis catus*, and a mosaic of closed and open foraging patches, allowing reliable assessment of spatio-temporal change in predation risk. We investigated the way such a change triggered individual prey decisions on where, when and how to perform routine activities.

**Results:**

Rabbit presence and behavior were recorded both day and night in patches with similar foraging characteristics, but contrasted in terms of openness. Cats, individually recognizable, were more active at night and in closed patches, in line with their expected higher hunting success in those conditions. Accordingly, rabbits avoided using closed patches at night and increased their vigilance if they did. Both day and night, rabbits increased their use of closed patches as compared to open patches in windy conditions, thereby probably reducing the thermoregulatory costs expected under such harsh environmental conditions.

**Conclusions:**

Overall, our data map the landscape of fear in this study system and indicate that prey habitat use and vigilance complement each other. Solely focusing on one or the other tactic may lead to erroneous conclusions regarding the way predation risk triggers prey decisions. Finally, future studies should investigate inter-individual variability in the relative use of these different types of complementary behavioral responses to perceived risk, along with the determinants and outcomes of such tactics.

## Background

Because predation risk varies in relation to space, time and individual ability to detect predators, behavioral responses developed by prey species to avoid being killed encompass individual decisions related to where, when and how to lead routine activities. Understanding what these decisions are, and what drives them, has been a major focus of behavioral ecology over the past decades (reviewed in [1, 2]). Accordingly, prey habitat selection, time budget and fine-scale anti-predator behaviors in relation to predation risk have been deeply investigated in the predator–prey interactions literature. Habitat selection in relation to predation risk is a widespread behavior in many prey taxa. In the southern Gulf of St. Lawrence, distribution of prey fishes were strongly related to risk of predation by grey seals (*Halichoerus grypus*), with distribution shifting into lower risk areas as predation risk increased over a 42-year period while non-prey species did not show similar changes in habitat use [3]. At finer spatial and temporal scales, the probability of being killed also shaped habitat selection in African ungulates [4] and migratory birds [5]. Predation risk also varies over time, and prey species have been shown to behave accordingly. When facing predation risk by raptors that exclusively hunt during daytime, rabbits (*Oryctolagus cuniculus*) mainly foraged during the night [6] but when fresh pellets of mink (*Mustela vison*), a nocturnal predator, are added in experimental plots, rabbits shifted their activities to the day. Temporal variation in predation pressure led to prey behavioral adjustments in several other taxa, including birds (e.g., [7]), fishes (e.g., [8]) and arthropods (e.g., [9]). Finally, for a given space and time, prey may decrease predation risk by adjusting fine-scale behavioral responses (i.e., at the scale of the body posture). For instance, in line with an increased predator detection probability for more vigilant individuals [10, 11], greater kudu (*Tragelaphus strepsiceros*) displayed longer vigilance bouts when lions (*Panthera leo*) were in the vicinity [12] and, when perceiving risk, impalas (*Aepyceros melampus*) decreased allogrooming probability [13], thereby avoiding a head posture impairing predator detection [14].

Although the above behavioral adjustments to perceived predation risk are generally well understood, our global picture of behavioral management of fear by prey remains fragmentary. This stems from the fact that only very few field studies considered these behaviors concomitantly [15–18], probably in part for historical reasons (habitat use and vigilance dealing with ecological and behavioral sciences, respectively [1]), although they are expected to be complementary [1, 19–21]. For instance, individuals may select more exposed foraging habitats if they increase vigilance. In this study, we simultaneously investigated in the same prey species population how spatial and temporal variation in predation risk triggered individual decisions of where, when and how to perform routine activities.

Designing studies allowing for adequate examination of these questions is complicated, as in most systems, the diversity of the predators to which the prey will respond and the heterogeneity and complexity of the habitats that can mediate the perceived risk lead to difficulties in identifying what safe or risky places, or times, are. Here, we took advantage of a simple system that allowed us to identify the main drivers of spatio-temporal patterns of risk: we focused on one prey, European rabbits (*Oryctolagus cuniculus*), that experience predation by feral cats (*Felis catus*) only, in a habitat with cover as the main and well-defined source of spatial heterogeneity in risk. The way cover shapes risk perception remains equivocal in the “landscape of fear” literature [1, 22], in part because the ratio between the contrasting obstructive (i.e., prevents the prey from seeing or escaping the predator) and protective (i.e., prevents the predator from seeing or attacking the prey) properties of cover, and thus the overall risk perception, is highly specific to a prey species, predator species and cover type [23, 24]. The characteristics of our system (namely, a single type of cover, used as a camouflage by a single stalk-and-ambush predator and acting as a physical barrier when escaping) allowed us to confidently consider cover items as a source of risk for rabbits, i.e., far more obstructive than protective (for more details, see [25]). Regarding temporal aspects, the way day/night succession shapes risk perception and concomitantly habitat selection and fine-scale behavioral responses is less investigated than spatial aspects in the literature [26]. Multi-predator systems may lead to complex patterns, both when explaining habitat use (e.g., [4, 27]), fine-scale behavioral responses (e.g., [28]) or both (e.g., [16]). In our single-predator system, the temporal pattern of risk was initially less certain that the spatial pattern: although a preliminary tracking study of 3 cats suggested that this predator was most active late afternoon [29], our field observations over the years rather suggested that cats were mostly hunting at night. Increased cats activity at night was later confirmed by our results (see “Results” section).

Having identified risky places and risky times, we recorded rabbit presence and behavior in safe (i.e., open) and risky (i.e., closed) patches during day and night and studied how rabbits combined time budgeting, habitat selection and vigilance to decrease risk. We predicted that rabbits should avoid situations where predation risk peaks or increase vigilance behavior in such circumstances while decreasing foraging (expected to shorten visual field given the head position) and resting, that could be performed in less risky circumstances (e.g., [30]) and may prevent rapid escape. Further, we expected open habitats, and, to a lesser extent, closed habitats, to be less attractive during windy days, both because increased predation risk through impaired hearing, smelling and seeing ability and because thermoregulation costs [2]. Hence, we also monitored daily wind intensity to investigate how this parameter impacted prey decisions and confirmed the expected “patch type” effect on wind speed. Finally, following previous studies reporting a common effect of group size on risk perception in prey [2], we recorded the number of conspecifics in the surroundings of the focal individual. Besides predation risk, the quality and quantity of resources are also expected to trigger both habitat selection and prey fine-scale behavior such as vigilance [31]. Therefore, we focused on patches of a single plant species and statistically confirmed that mean plant height did not differ according to habitat openness.

## Methods

### Study area

Our study was conducted in the Pointe Morne area (49°220 S, 70°260 E) on the Kerguelen archipelago, from November 2016 to January 2017. To our knowledge, this is the only area on the archipelago with both rabbits and cats, displaying such particular cover items. The average annual temperature on Kerguelen is 4.5 °C (with 116 frost days on average per year), the mean annual wind speed is 9.8 m/s (wind speeds greater or equal to 16 m/s occur on 300 days/year) and precipitation occurs on an average of 285 days/year (of which 23 have a total of greater than 10 mm) [32].

Introduced by sailors during the nineteenth century, rabbits are now widespread throughout the archipelago [33]. Domestic cats were introduced in 1951 to control invasive rodents and rabbits at the research station of Port-aux-Français. Cats are now widely distributed over the main island. Although predation by brown skua (*Catharacta lonnbergi*) on sick or young rabbits occurs, studies suggest that predation pressure experienced by rabbits in our study area is by far mostly due to cats [33, 34].

### Patch characterization

We focused on a ca. 100 by 700 m area, where cover was only provided by mounds, less than 2 m high and formed of earth and roots and covered by the perennial herb *Acaena magellanica* (Rosaceae). The space between the mounds was covered by *Acaena magellanica*, *Poa annua* and bare ground/rocks (more details in [25]). Following the procedure explained in [25], we expected over 150 different individual rabbits to forage on our studied patches in the study year.

We defined a ‘‘patch’’ as a circular area with a 2 m diameter, covered by *Poa annua*, whose center was at least 20 m away from the center of another patch. *Poa annua* is a highly nutritious alien grass that represents most of rabbit diet in our study area (over 90% of the plant fragments found in fecal pellets at the time of the year our study took place, i.e., summer, [35]). Focusing on patches covered by a single plant species prevented important changes in forage quality between patches.

### Data collection

In a first step, we searched the study area for patches, which numbered 30. We then calculated the unobstructed area around each patch, which represents the overall area from the center of the patch to the surrounding mounds (higher than 20 cm, i.e., capable of hiding an ambushing cat from a rabbit, even in an upright posture, and of hiding a rabbit from a cat, unless the rabbit was in an upright posture), following procedure detailed in [25].

In a second step, we selected the 7 patches with the largest unobstructed area (thereafter referred to as “open” patches) and the 7 patches with the smallest unobstructed area (thereafter referred to as “closed” patches) and deployed a camera-trap (Reconyx PC 900 or PC 950) in those patches from the 29th November 2016 to the 13th January 2017. Camera traps have been used previously for fine-scale behavioral studies of prey facing risk (e.g., [36, 37]). For each patch type, one camera did not take any picture (malfunction or destruction by an animal) and one ran out of batteries before the 13th January (i.e., on the 20th December and 4th January) but their data were included in the analyses when relevant (see below).

Camera traps were set up to take one picture per hour (thereafter referred to as “time lapse” setting) as well as 10 pictures (1 per second) each time an animal’s movement triggered the motion detector (including at night) with no quiet period (thereafter referred to as “motion detector” setting) (i.e., similar design as in [37]). We used pictures from the time lapse setting to investigate variability in patches frequentation and pictures from the motion detector setting to study variability in rabbit behavior. We delimited patches with small rocks, which allowed us to visualize more clearly on pictures whether rabbits (i.e., their four legs) were inside a patch.

We assessed whether plant height (i.e., forage availability) and wind speed differed between patch types. In each patch, we measured plant height at 15 random location points, at the beginning of the study. Plant height did not differ between patch types (accounting for spatial autocorrelation in model residuals—see “Statistical analyses” below, LR = 0.24, *df* = 1, p = 0.62). We also measured, in each patch, the average wind speed during 1 mn at 20 cm elevation with a hand anemometer, once a day during 8 days (13th January–20th January, i.e., outside the recording period to avoid disturbance). Wind speed was 76% higher in open patches (average speed: 2.5 m/s and 4.4 m/s in closed and open habitats respectively; LR = 40.04, *df* = 1, p < 0.001). For the main analyses (29th November 2016–13th January 2017), climate data were available from the meteorological station of Port-aux-Français research station, located about 15 km away from the study area.

Finally, to later confirm that camera-trap data could correctly estimate rabbit frequentation of patches, we removed all fecal pellets from patches at the beginning of the study, and counted the number of pellets at the end. Focusing on patches with complete recording (n = 10), the number of time lapse pictures with a rabbit was strongly related to the total number of pellets collected at the end of the session (r = 0.92, LR = 66.60, *df* = 1, p < 0.001). Pellet quantity has been proved to be a reliable method to assess rabbit abundance in other systems [6, 38].

### Data coding

As only few time lapse pictures included rabbits (225/11,909) and because we were interested in a night/day effect on rabbit presence, we coded rabbit presence at the day/night scale rather than at the hour scale. For each date and each period (day or night), the presence/absence variable was coded “1” if at least one rabbit was seen, “0” otherwise. Most pictures (211/225) displayed only one rabbit.

To code behavioral data acquired using the “motion detector” setting, we first excluded all the pictures displaying other animals than rabbits, rabbits outside the patch or young rabbits (whose individual decisions may differ from adults’, e.g., [2, 39], and who were too rare to be analyzed separately). We then randomly selected pictures taken with at least a 5 mn interval. This led to a total of 1289 pictures. We then screened each of these pictures and attributed one of the behaviors described below. When several rabbits were present on the same picture, we selected the individual closest to the center of the patch. In some occasions, we screened the picture(s) before and/or after the focal picture, when included in a sequence, to better interpret the behavior. We coded the following behaviors: (i) foraging (head down in the grass), (ii) vigilance (immobile with a head-up posture and erected ears; this always occurred during moving or foraging bouts) and (iii) resting (non-foraging immobile individuals with a contact between the stomach and the grass, e.g., lying down). Other behaviors (such as grooming or socially interacting) were too rare to be analyzed separately and functionally too different from one another to be analyzed simultaneously.

We recorded cat visits to patches using “motion detector” pictures. Cats were individually recognizable on pictures through their coat-color patterns, ranging from black to black and white [40]. Four different individuals were identifiable on the pictures. We did not observe cats on “time lapse” pictures.

### Statistical analyses

For the analysis of both rabbit presence and behavior, we used models with a binomial response variable (with “1” for presence of a rabbit or occurrence of a specific behavior and “0” otherwise) and, as explanatory variables, we considered: the period (thereafter, “Period”, day or night), the patch type (thereafter, “Obstruction”, closed or open), the wind speed (at the regional scale, i.e., available from the meteorological station of Port-aux-Français research station; thereafter, “Wind”, with “0” when below the average—i.e., 10.04 m/s—and “1” when above) and, for the analysis of rabbit behavior only, the presence of other rabbits on the picture (thereafter, “Other rabbits”, with “0” if no other rabbit, “1” otherwise, with 76% with a single extra individual). For the analysis of rabbit behavior, as we were mostly interested in investigating a period and patch type effect, as we had no particular reasons to expect an interaction between wind speed and group size, and, finally for sample size/balanced design purposes, we first ran a model with Period, Obstruction and Other rabbits including all the interactions and then the same model but substituting Other rabbits by Wind speed. We used spaMM package [41] in R 3.1.2 [42], to account for both spatial (using GPS coordinates of each patch) and temporal (using Julian date) autocorrelation in model residuals. We further included patch identity and date as random terms to account for non-independence between observations performed in the same place and at the same date. We selected the final model by fitting the complete model including the interaction and removing each term successively. The significance of each term was determined by assessing the change in deviance (i.e., Likelihood Ratio Test) against a Chi squared distribution, with the appropriate degrees of freedom. We used the same approach to test whether cats were more frequently observed at night and in closed or open patches, including cat identity in the models to control for non-independence between observations of the same individuals, and for the analyses previously described (i.e., the relationship between the number of pictures displaying at least a rabbit and the total number of fecal pellets, the Obstruction effect on wind speed and the Obstruction effect on plant height).

## Results

### Factors related to cat presence

In line with our personal observations, cats visited patches much more frequently at night (LR = 18.02, *df* = 1, p < 0.0001). Our data also revealed that they visited closed patches more often than open patches (LR = 5.67, *df* = 1, p = 0.02), while the interaction between both factors was not significant (LR = 2.15, *df* = 1, p = 0.14).

### Factors related to rabbit presence

**Fig. 1:**
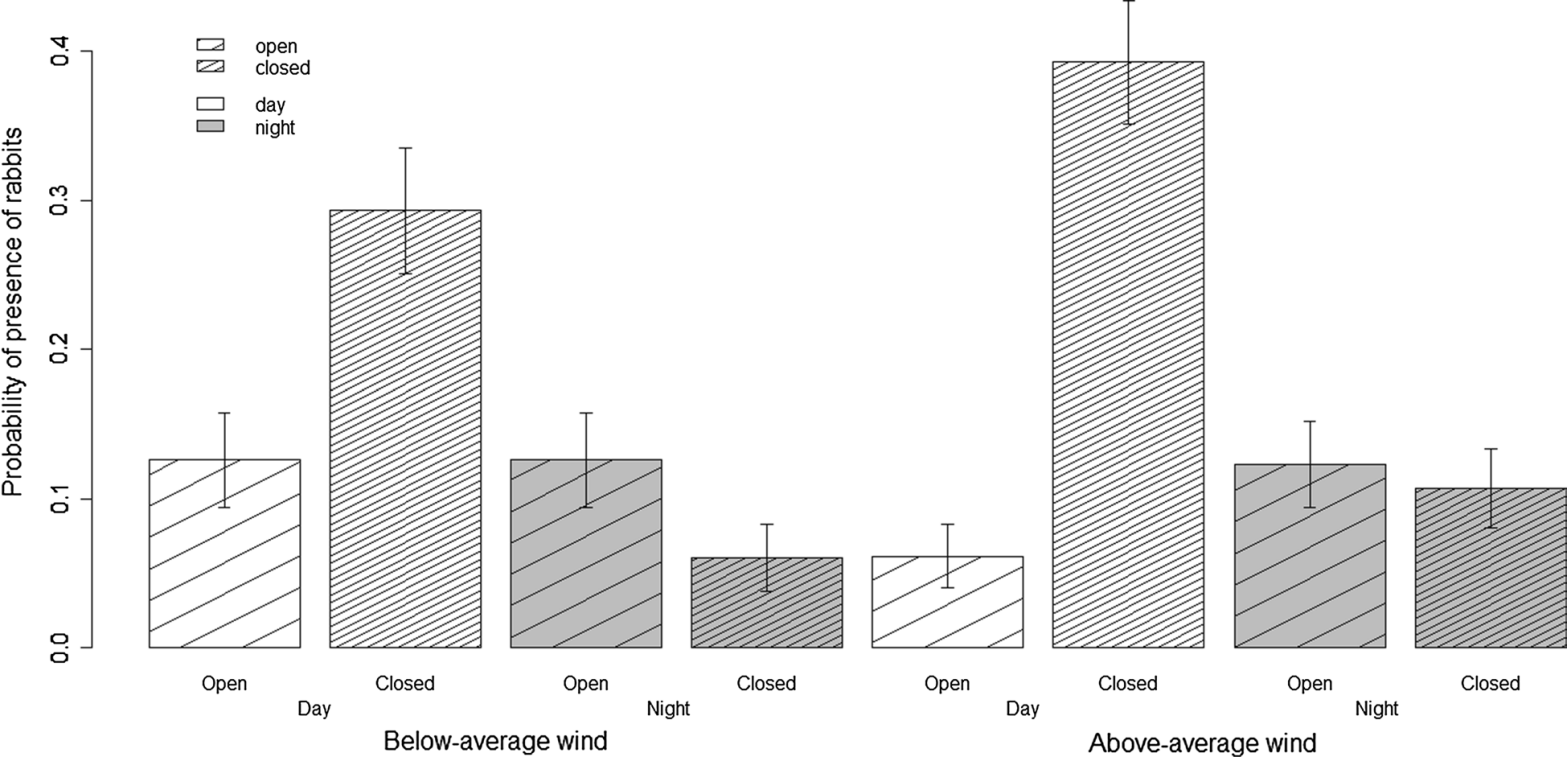
Probability of presence of rabbits (± SE) according to habitat openness (obstruction), day/night (period) and wind intensity (wind). All patches and dates of observation are pooled. In the analyses, spatial and temporal autocorrelation in model residuals are accounted for and patch identity and date are included as random terms (see text)

The probability of rabbit presence was related both to the interaction between Obstruction and Period and to the interaction between Obstruction and Wind (Table 1; intercept = − 1.09 ± 0.35; coefficients: open:night = 2.26 ± 0.40, open:above average wind = − 0.81 ± 0.39, night = − 1.85 ± 0.27, open = − 1.43 ± 0.53, above average wind = 0.59 ± 0.26), indicating that rabbits avoided using closed habitats at night as compared to daytime and increased their use of closed patches as compared to open patches in above average wind conditions both night and day (Fig. 1).

**Table 1:** Model selection for rabbit presence probability

Explanatory variables	LR	*df*	p-value
Obstruction × period × wind	0.32	1	0.57
Obstruction × period	33.73	1	< 0.001
Obstruction × wind	5.21	1	0.02
Period × wind	0.93	1	0.33

Final model: Presence = obstruction × period + obstruction × wind + obstruction + period + wind

### Factors related to rabbit behavior

**Fig. 2:**
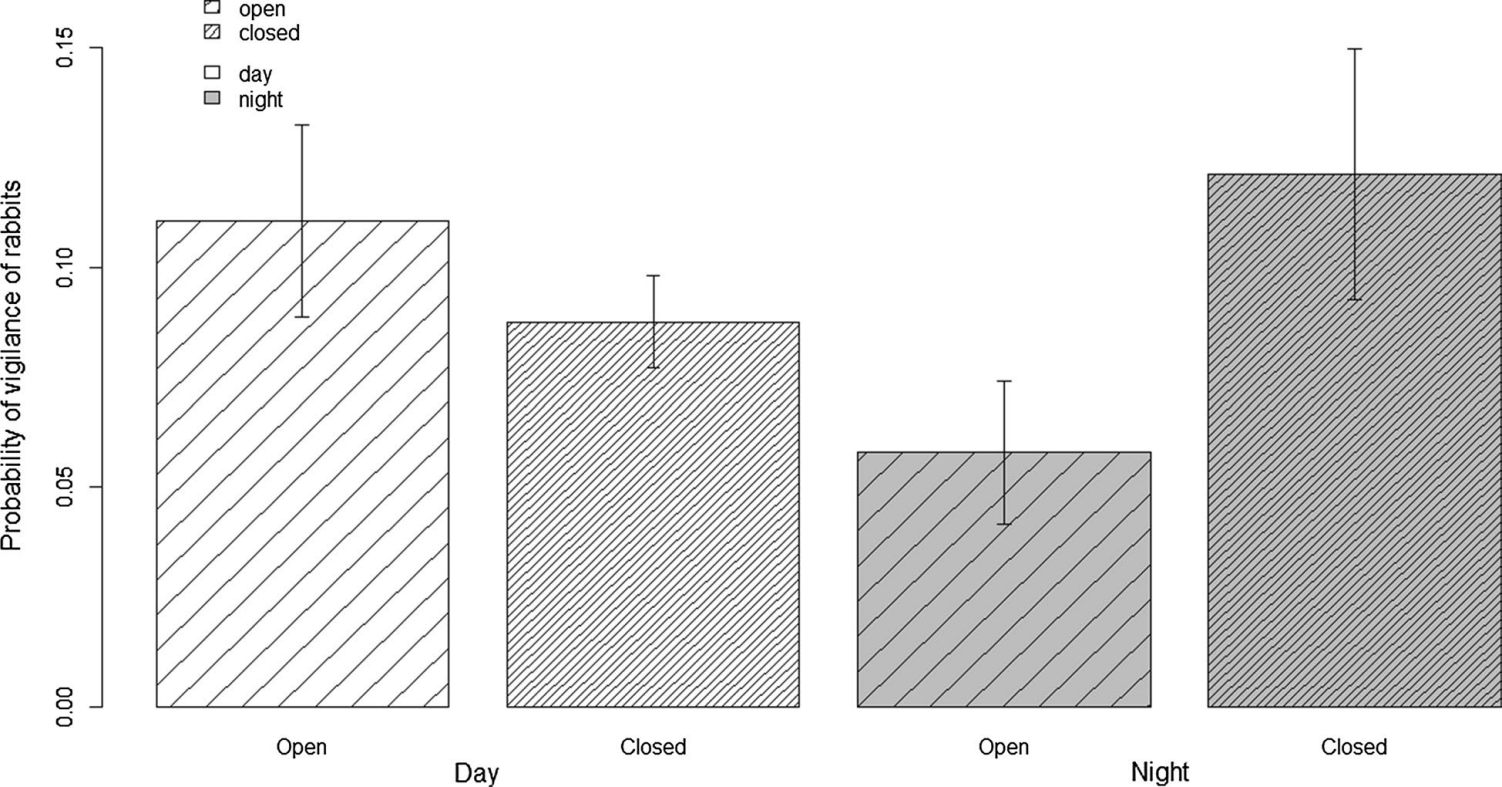
Probability of vigilance of rabbits (± SE) according to habitat openness (obstruction) and day/night (period). All patches and dates of observation are pooled. In the analyses, spatial and temporal autocorrelation in model residuals are accounted for and patch identity and date are included as random terms (see text)

In the first set of models including Obstruction, Period and Other rabbits, the probability of vigilance behavior was related to the interaction between Obstruction and Period (Table 2; intercept = − 2.39 ± 0.33; coefficients: open:night = − 1.03 ± 0.50, night = 0.46 ± 0.31, open = 0.19 ± 0.34), indicating in particular that at night, rabbits increased vigilance behavior in closed habitats as compared to open habitats and to a lesser extent, to daytime (Fig. 2). Foraging or resting probability were not impacted by the explanatory variables we considered (Tables 3 and 4). In the second set of models, substituting Other rabbits by Wind led to similar results, meaning in particular that we reported no effect of Wind on vigilance, foraging or resting probability.

**Table 2:** Model selection for rabbit vigilance probability

Explanatory variables	LR	*df*	p-value
Obstruction × period × other rabbits	< 0.001	1	0.99
Obstruction × period	4.69	1	0.03
Obstruction × other rabbits	0.05	1	0.82
Period × other rabbits	1.37	1	0.24
Other rabbits	1.84	1	0.17

Final model: Vigilance = obstruction × period + obstruction + period

**Table 3:** Model selection for rabbit foraging probability

Explanatory variables	LR	*df*	p-value
Obstruction × period × other rabbits	3.39	1	0.06
Obstruction × period	1.80	1	0.18
Obstruction × other rabbits	0.05	1	0.82
Period × other rabbits	0.18	1	0.67
Other rabbits	0.09	1	0.76
Obstruction	1.84	1	0.17
Period	1.62	1	0.20

Final model: Foraging = constant

**Table 4:** Model selection for rabbit resting probability

Explanatory variables	LR	*df*	p-value
Obstruction × period × other rabbits	0.72	1	0.40
Obstruction × period	0.99	1	0.32
Obstruction × other rabbits	0.89	1	0.35
Period × other rabbits	1.00	1	0.32
Other rabbits	0.16	1	0.69
Obstruction	0.03	1	0.87
Period	1.26	1	0.26

Final model: Resting = constant

**Fig. 3:**
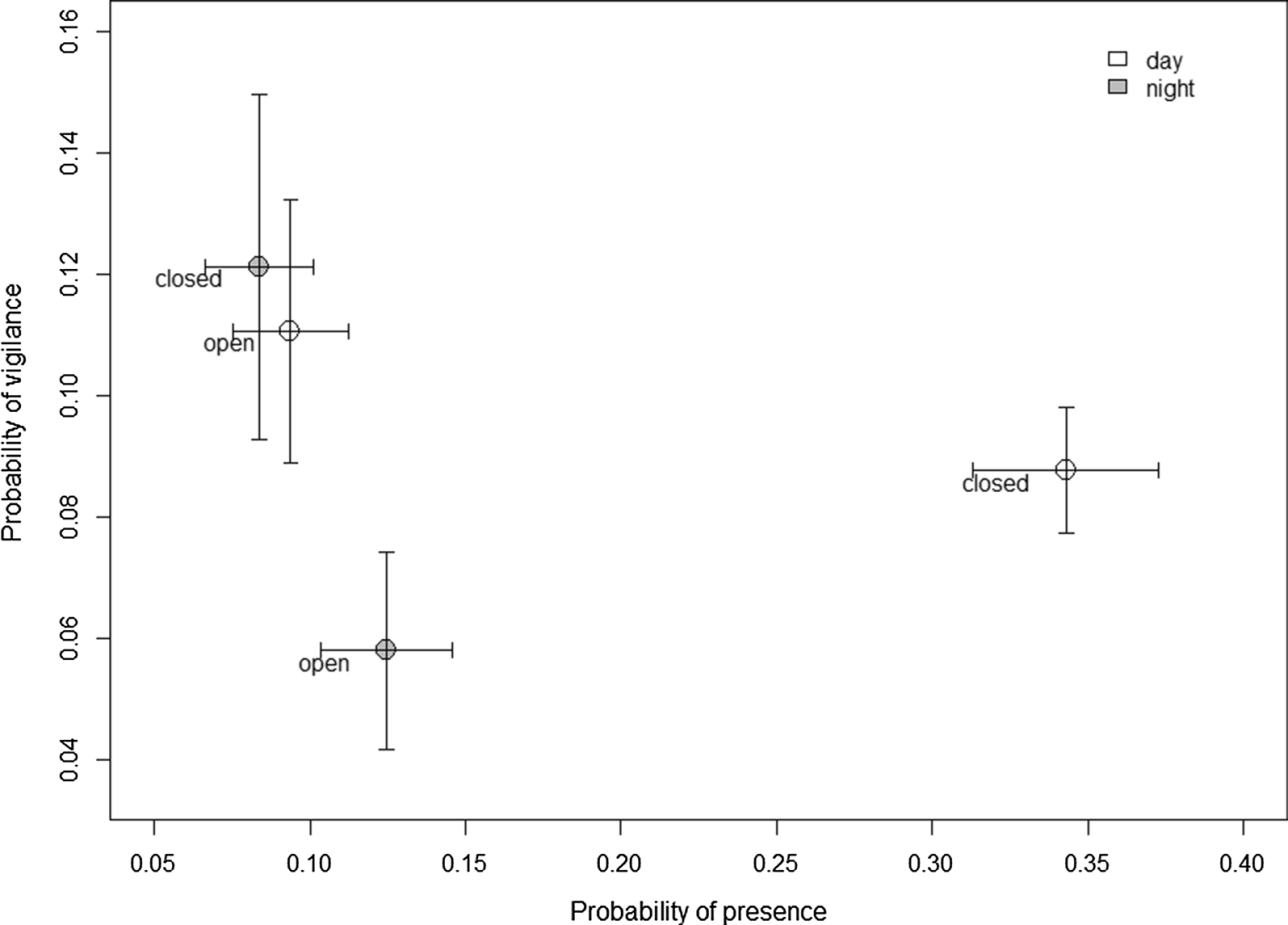
Probability of vigilance (± SE) in relation to probability of presence (averaged for both wind condition, ± SE) in rabbits, according to habitat openness (obstruction) and day/night (period). All patches and dates of observation are pooled. In the analyses, spatial and temporal autocorrelation in model residuals are accounted for and patch identity and date are included as random terms (see text)

On Fig. 3, we plotted vigilance probability in relation with presence probability, for both levels of Obstruction (open and closed habitats) and Period (day and night) (averaged for both Wind conditions) in order to show how anti-predator tactics complemented each other.

## Discussion

Studies investigating how spatio-temporal variation in risk is dealt with by prey, simultaneously considering several anti-predator behaviors, are rare. Such studies are however required to understand how anti-predator behavioral adjustments can compensate for each other and thus to give a more complete picture of the way in which prey cope with predation risk [21]. We addressed this knowledge gap by studying a simple prey—predator—habitat system that allowed us to make reliable predictions regarding spatial and temporal variation in predation risk. In line with their expected higher hunting success in those conditions, cats were more active in closed patches and at night. Prey could decrease risk of being killed by avoiding risky situations and/or by increasing their ability to detect approaching predators when in risky situations. Our data suggest that in our system, habitat use and vigilance behavior complement each other (Fig. 3). Rabbits strongly reduced their use of closed patches during nighttime as compared to daytime, thereby avoiding the cumulative effect of increased temporal (more cats at night) and spatial (more cats in closed areas) predation risk. At night, i.e., at risky times, rabbits compensated for further increased predation risk when in closed habitats, i.e., in risky places, by investing more heavily in vigilance. We underline the importance of the day/night comparison of rabbit behavior when identifying the role of predation pressure in this system. We therefore concur with previous authors stressing the importance of nighttime observations [43]. Clearly, the “landscape of fear” [22] and the “schedule of fear” [16] are part of the same story, as shown by previous studies in rabbits (e.g., [44, 45]) and other taxonomic groups [26, 46].

### Spatio-temporal variation in rabbit presence

Changes in habitat selection in prey according to time of the day in a context of predation are often reported [26, 43, 46, 47]. In rabbits, previous results have reported that individuals preferentially fed closer to cover during the day (hiding from birds of prey) than at night (avoiding stalking carnivorous mammals) [45]. Wind intensity also impacted rabbit habitat selection in our study area: the use of closed patches increased both during daytime and nighttime in windy situations. In line with previous studies reporting increased difficulty in detecting an approaching predator in windy situations [2] and with our data showing that closed habitats offered greater protection against wind, we can speculate that rabbits selected habitats offering better detection ability of potentially more abundant predators. Hence, decreased cat detectability in open patches through wind effect would outweigh increased cat encounter probability in closed patches, leading rabbits to increase their use of the latter. Thermoregulation aspects probably also matter under such harsh climatic conditions. The way wind shapes prey detection ability and thermoregulation in cats also requires investigation. Habitat selection according to wind in a predation–prey context is seldom documented [48]. Underlying factors related to habitat openness include olfactory [49] or visual [50] cues, or more complex patterns [51].

Beside the interactions between patch type and period (day/night) and between patch type and wind speed in explaining rabbit presence, rabbits were overall more common in closed patches (test of patch type alone: LR = 4.42, *df* = 1, p = 0.04), and this was driven by daytime habitat selection. Although closed patches are expected to be more risky, even during daytime, the fact that rabbits still use them is not surprising given that a compromise between patch safety and resource availability is expected to arise soon or later. However, the overall preference for riskier closed patches might appear surprising at first sight, especially in the light of previous results on the same population, reporting more pellets in open patches, which was interpreted as a result of lower predation risk [25]. Two main hypotheses may explain this apparent contradiction between 2014 (the year considered in [25]) and 2016 (this study). First, because our data revealed that rabbits avoided using open patches under windy conditions, we hypothesized that the 2016 session was windier than the 2014 session. We thus compared the daily wind speed during the camera trap session in 2016 with the daily wind speed 60 days prior to pellets collect in 2014. We chose 60 days as most of the pellets disappeared within this lapse of time (authors’ unpublished data). We found no differences (W = 1357, p = 0.78). Secondly, we questioned predation pressure. In 2015, the Réserve Naturelle des Terres Australes Françaises started to control the cat population in the study area (18 individuals captured in July–September both in 2015 and 2016, [52]), that succeeded in reducing the population density by more than three-fold (author’s unpublished data). The difference in overall habitat selection by rabbits between both sessions could thus be the result of decreased predation pressure, with closed patches being less risky than previously, in particular during the day were only few cats are around, while offering greater protection against wind. This speculation would echo with previous results on rabbit behavioural changes following predator control [53].

### Spatio-temporal variation in rabbit fine-scale behavior

Identifying predation risk as an explanation for an effect of time of the day on vigilance is not always easy as time of the day may be confused with other factors, such as habitat type or group size, both well-known drivers of vigilance [2]. Here, despite a clear effect of the interaction between patch type and period (day/night) on rabbit presence, we were able to observe rabbits in both habitats during both daytime and nighttime. Moreover, there was no interaction between patch type and period (day/night) in explaining variation in the number of other rabbits (LR = 0.31, *df* = 1, p = 0.58), although group size was previously related to predation risk in rabbits (e.g., [54]). Hence, in closed habitats, the increased vigilance at nighttime as compared to daytime is probably the result of an increased predation risk perception. Reviewing the literature, Beauchamp [2, 26] showed that vigilance was typically lower at night for birds and mammals, although exceptions occurred ([1]; for a more general discussion of how vigilance tactics should vary with ecological conditions, see [55]). Lower vigilance at night may be the result of decreased predation risk or decreased utility of scanning in the dark [26]. In our system, cats were more active at night, leading to an actual increased predation risk, in particular in closed habitats regarding increased visitation rate by cats (and expected increased hunting success). Regarding the utility of scanning at night, beside visual aspects, vigilance posture may also allow a better olfactory (and perhaps auditory, e.g., [56]) detection of an approaching predator. Springbok (*Antidorcas marsupialis*) also increased vigilance at night, i.e., when their predators were more active [57]. Furthermore, we suggest that habitat characteristics matters in the “scanning utility”–“light level” relationship. The expected decrease in the utility of nighttime scanning as compared to daytime scanning might be less pronounced in closed habitats as cover may limit the visual field anyway, while visual limitation through decrease luminosity is more expected farther away from the focal animal. In open habitats, the strongly decreased vigilance at night might conversely be the result of an actual decreased utility of scanning in the dark given that reduced surface area is visible, in particular under relatively safe circumstances as compared to closed habitats. Overall, this leads to the reported increased day/night absolute difference in vigilance levels in open as compared to closed habitats. Still, the high absolute level of vigilance in open habitats during daytime (i.e., under low expected predation risk) deserves more investigation. Although we did not report any wind effect on vigilance probability, maybe as a consequence of relatively high mean daily wind speed in our study area, a possible explanation is an impact of wind in more exposed open patches. Contrary to the inefficiency of scanning in the dark suggested above, increasing vigilance in windy situation may allow to compensate for decreased visual, olfactory and auditory performance [2].

## Conclusions

Overall, our data map the landscape of fear in this study system and indicate that prey habitat use and vigilance complement each other to reduce risk perception. Solely focusing on one or the other tactic may lead to erroneous conclusions regarding the way predation risk triggers individual prey decisions. For instance, without data on vigilance behavior, showing that individuals facing risky times and places increased their investment in vigilance, our results would have suggested a stronger difference between individual tactics in terms of the magnitude of exposure to predation than the difference we actually report.

Unlike cats, rabbits were not individually recognizable. Hence, whether some individuals track spatio-temporal variability in risk and adjust their habitat use accordingly while others preferentially rely on vigilance behavior to buffer change in predation pressure in a given place according to time remains an open question. If such inter-individual variability in the relative use of different behavioral responses to risk perception exists, investigating associate differences in mortality risk but also in stress level or foraging success, i.e., in risk effects sensu [58], would be stimulating. Similarly, identifying factors shaping such inter-individual variability would also be of interest. Consistent behavioral differences between individuals are indeed now well established in many taxa (e.g., [59]) and previous studies have revealed such behavioral profiles in prey (e.g., [60]). Other hypotheses, including an effect of social rank, with subordinate individuals being forced to suboptimal decisions [61], could explain a potential inter-individual variability in the use of anti-predator tactics.
